# Open Logistics: Blockchain-Enabled Trusted Hyperconnected Logistics Platform

**DOI:** 10.3390/s22134699

**Published:** 2022-06-22

**Authors:** Ali V. Barenji, Benoit Montreuil

**Affiliations:** 1 School of Industrial and Systems Engineering, Georgia Institute of Technology, Atlanta, GA 30332, USA; benoit.montreuil@isye.gatech.edu; 2 Physical Internet Center, Supply Chain & Logistics Institute, Atlanta, GA 30332, USA; 3 Coca-Cola Chair in Material Handling and Distribution, Atlanta, GA 30332, USA

**Keywords:** open logistics, blockchain, supply chain 4.0, hyperconnected, asset sharing, trust-ability

## Abstract

The digitalization and adoption of advanced technologies in supply chain and logistics not only change the business model but also transfer logistics infrastructure to a service-oriented architecture and introduce new avenues concerning supply chain 4.0 (SC4.0). Sharing logistic assets between various businesses leads to improving logistics work, enhancing work productivity, and reducing logistics expenses and environmental impact. However, due to the lack of a secure, trustworthy, and open sharing platform, the companies are not willing to rely on sharing economics. Aiming to improve trust-ability, openness, and interoperability in the SC4.0, this paper presents a blockchain-enabled hyperconnected logistics platform. Firstly, the Open Logistic platform (OL) is proposed, and the key characteristics of this platform are explained. Secondly, the concept of proof of delivery (PoD) based on smart contracts is defined and developed to explore its rule-based management and control among the dynamic assets sharing. Thirdly, the Blockchain asset sharing service is designed and discussed in the context of asset sharing. Fourthly to evaluate the feasibility of the proposed platform, a simulation environment is developed, and OL is implemented based on the case study.

## 1. Introduction

The potential of logistics, which are vital contributors to most industries and countries, depends on their power to react to their customers’ expectations while preserving a competitive advantage in their marketplace. A disruption in one company can affect multiple companies in the network. When an unplanned and unanticipated event occurs, the normal flow of goods is disrupted, which has urgent consequences for the costs and profitability and long-term consequences for the brand image and customer satisfaction level. Therefore, the performance of supply chains is highly sensitive to risks in the network [1]. This supply chain problem was more apparent during COVID-19. Companies and transportation hubs have been closed, new safety protocols implemented, the movement of employees restricted, and customer needs and demands have changed. No region or industry has been immune to these effects.

To overcome this challenge and minimize the risk of supply chains, some enterprises invest more in the hubs, resources, digitalization, inventories, health workers, safety, etc. [2]. Another solution is sharing economics which opens doors to other retailers or logistic service providers. For instance, Walmart recently announced the launch of a delivery service called GoLocal, which will carry goods from other local retailers to consumers [3].

Sharing economy is a key solution to achieving fast, high-quality service. Besides, it could be the best solution to the predicated and unpredicted risk of supply chains. The sharing economy has already transformed several industries through the popularity of apps such as Mobility and Hospitality. The sharing economy will assist logistics, and it allows all contributors to share secure costs, enabling businesses to make several slighter investments rather than a single large investment, which would lead to rationalizing logistics work, improving work efficiency at delivery points, and cutting transport costs and environmental impact, compared with the conventional systems [4,5].

The need for sharing logistics has never been so imperious, especially since the growth of risk and uncertainty has become a serious problem. To quench their thirst for the fast delivery of parcels, small, mid-size enterprises (SMEs), and large enterprises are turning to sharing resources. Generally, sharing logistics is recognized as a significant business strategy by [6], in which supply chain entities work collaboratively on a short-term basis to deliver parcels, advanced by digitalization which is the mainstay of supply chain 4.0.

In this respect, researchers expand the concept of open logistics and manufacturing as sharing assets, services, spaces, and workers between business processes across internal or external partners in the value chain [7,8]. Soon Hong Min et al. [9] explained that the sharing economy has been referred to as the driving force behind effective supply chain management and may be the best core capability for overcoming the risks. However, the sharing economy could dramatically increase the risk of malicious attacks, and, accordingly, induces trust problems between multiple hyperconnected service providers. The current challenges of the sharing economy for supply chains are explained as follows.

- **Information security** is invariably the main apprehension in effective collaboration. The supply chain industry is presently experiencing its own digital transformation in the form of SC4.0, where cyber-physical systems combine the physical world and digital networks to effect change. However, SC4.0 hosted a complete novel variety of safety and security issues. These security issues range from simple threats which can be easily mitigated or even ignored, to very complex threats that can render the whole system unusable [10].
- **Trust and Transparency.** Mutual trust and protection must be maintained between all parties in the sharing economy to guarantee a high quality of service, mitigate disputes, handle payments securely and, most importantly, encourage engagement of users and service providers. For example, in the business-to-business sharing logistics model, counterfeits and payment disputes are major issues [11].
- **Liability and Insurance.** The sharing logistics can be fraught with risks and liability. There is a risk that the goods or services are being shared with a lower standard quality than expected or could cause physical damage to the service requester. For the service provider, the highest risk is theft, loss, or damage. Since the platforms do not own the assets, there is little incentive initially for the platform providers to ensure goods or services. Today, users and service providers must typically ensure themselves and ask their insurers to find the best individual solution.
- **Interoperability and data sharing problems.** There is a shortage of sharing asset and resource protocols and standards to integrate, interoperate and share the data along the supply chain process [12]. Many interoperability and data sharing problems exist between the resource share entities. Traditionally, it has been addressed with “point-to-point” solutions and standards [13]. However, it can hardly be widespread by the multiple collaborative parties due to the trade secrets, regional policies, etc.

Recently a novel peer-to-peer technology has been introduced for manufacturing and supply chain industries as blockchain, which can omit third parts and improve the security of the system. In this respect, a trustable collaboration platform is proposed based on blockchain and fog computing to reduce the gap between designers, customers, and manufacturers aiming to introduce an open manufacturing concept that causes interoperability and data sharing problems to improve [14]. Blockchain-based distributed logistics platform is introduced with the power of distributed IoT nodes and offers an alternative approach to dealing with the complexity of modern supply chains by breaking them into smaller, functionally independent parts [15]. Abir EL Azz et al. [16] proposed the implementation of blockchain and smart contracts with the information hiding technique to enhance the security and privacy of data communication in the healthcare supply chain. Also, Tarun Kumar Agrawal et al. [17] proposed a blockchain-based traceability framework for the textile and clothing industry. It is obvious that integrating blockchain technology into the logistics could be a potential solution. This paper attempts to raise the concept of Open Logistics (OL), a Blockchain-enabled, trustable hyperconnected logistics platform.

In this paper, we propose an OL platform to improve trust between parties and data transparency which is the main factor in achieving trustable assets and resource sharing in the sharing economy. Following the OL platform architecture, a practical roadmap is provided for the parcel logistics to achieve blockchain design, development, application, and evaluation. Moreover, three core enabling components are presented: (1) Network structure and blockchain-based software-defined networking layer, (2) Smart contract-based proof of delivery, and 3) Blockchain asset and resource sharing service.

This paper contributes to the logistic industry in the following three aspects. (1) It presents a blockchain-enabled sharing assets framework and its mechanism to realize a secure, interoperable, and feasible cooperation mode. (2) It provides a versioning smart contract-based concept to explore the PoD concept and control among the assets, workers, and materials, as well as the parties. (3) It develops an open logistics platform that could be used in existing paradigms.

The rest of this paper is organized as follows. Section 2 presents a review of the related research. Section 3 presents the overall architecture of the open logistics, as well as its core components. Section 4 describes the main components and mechanism of open logistics. Section 5 discusses the case study and implementation of open logistics. Section 6 covers the results and discussion, and Section 6 provides the conclusion and recommendations for further research.

## 2. Literature Review

To draw a clear distinction between this paper and previous literature, we reviewed the publications about collaboration, share logistics, and blockchain in collaborative platforms.

### 2.1. Sharing Logistics

Sharing logistics is the future (and the now) of successful, risk-managed logistics and supply chain management. Today, sharing means that companies work together through a neutral third party to segregate the data and confidential information. There is no direct access to confidential information between parties in that field. This is a broad perspective because it can have broad collaboration between different parties who have different suppliers. In the sharing logistic economy, parties work together to serve a common customer, or as a customer, bundling competing suppliers (i.e., truck companies) into one truckload to maximize utilization, etc. [18]. Yong Wang et al. [19] proposed eco-packages based on a time-space network for pickup and delivery sharing problems. They minimized the total operational costs by forming collaborative alliances and allocating trucking resources based on time-space network properties.

The impact of existing works in the sharing logistics economy is tremendous and has some merits in achieving collaborative and hyperconnected logistics. However, sharing logistics is not just sharing data; it combines sharing physical resources and data. In addition, most of the proposed paradigms are based on a centralized environment, though some are still faced with big challenges related to security and transparency. So, trust, transparency, and secure information sharing are the main challenges behind existing solutions.

### 2.2. Blockchain-Based Sharing Platform

A blockchain is a digitally distributed, decentralized ledger existing across a network. Initially, it is a fundamental technology for supporting the digital currency, such as bitcoin. Blockchain is a distributed data structure (ledger), that has risen as a promising technology. Each block typically contains a cryptographic hash of a previous block, a timestamp, and transaction data. It provides a secured and trusted data storage and computing solution. Moreover, blockchain creates a new type of distributed and peer-to-peer communication network. It helps users conduct their data transactions without any third party or with transparency and traceability [20]. It can provide a trustworthy computing distribution structure for the parties to exchange information and share services. The main principles of blockchain are named as follows; distributed database, peer-to-peer transmission, transparency, irreversibility, and computational logic [21]. Haoye Chai et al. [22] proposed a consortium blockchain-based resource sharing paradigm in the internet of vehicles, in which the resource sharing interactions are encapsulated as transactions and recorded by Roadside Units (RSUs). Moreover, a lightweight consensus mechanism named Proof-of-Reputation is proposed to reduce computational power consumption and motivate vehicles involved in resource sharing. Zonggui Tian et al. [23] provided viewpoints to help advance the discussion and motivate additional practice and research related to sustainable urban logistics and blockchain technology. Qi Yao et al. [24] integrated blockchain technology to solve the logistic traceability problems of the agricultural product, a dual-storage model of Blockchain + IPFS (InterPlanetary File System) intended to reduce the storage pressure of the blockchain and realize efficient information queries. Syed Abdul Rehman Khan et al. [25] studied the role of blockchain in the circular economy to enhance organizational performance. The questionnaire-based study was applied to show the possible advantages of blockchain technology and green information systems to sustainable supply chain practices [26]. Most recently, a blockchain-based tracking and tracing system for pharmaceutical products was designed and developed [27].

These studies provided some valuable insights into the application of blockchain in sharing logistics. Nevertheless, the specific computing and network architecture of blockchain-enabled sharing logistics platforms remains to be explored. Therefore, it is urgent to develop a trustworthy and secure computing architecture to transparent and trustable mechanisms of resource sharing in collaborative logistics.

## 3. The Architecture of the Open Logistics

**Figure 1:**
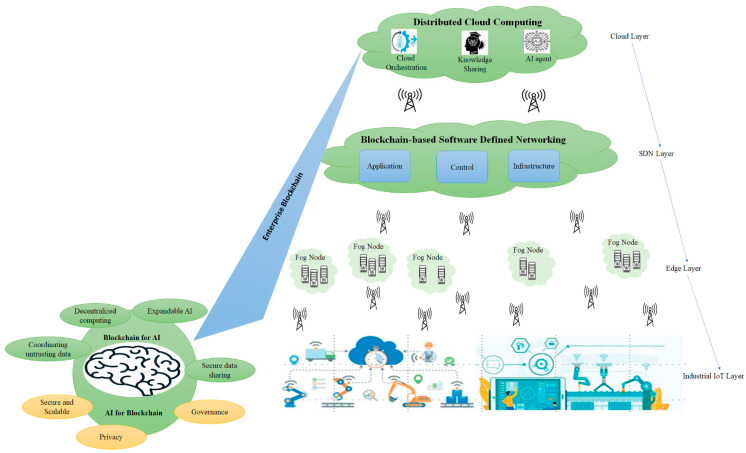
Layers and modular-based open logistics architecture.

This section presents OL architecture, working process, and enterprise blockchain network structure for OL. The architecture is shown in Figure 1. It contains four layers, including distributed cloud layer (DC), Blockchain-based Software-Defined Networking (BSDN) layer, edge layer, and industrial IoT device layer (IIoT), which are connected to physical resources. In addition, these four layers connect with the enterprise Block Chain Network (eBCN). DC layer provides data storage, computing power, and blockchain infrastructure. The edge computing layer is responsible for connecting and providing limited computing power to industrial devices to provide real-time communication, and the IIoT layer directly connects with industrial devices and offers a direct bridge between the edge computing layer and industrial devices. BSDN is responsible for delivering the cooperation of different network devices. The blockchain network provides a peer-to-peer network, decentralization, trust, and governance to OL. Therefore, the OL accelerates the development, governance, and operation of a multi-institution business network. Each layer and component is defined as follows.

### 3.1. Distributed Cloud Layer

The DC layer has geographically dispersed infrastructure that primarily runs services at the network edge. This is different from the theoretical cloud model that relies on a centralized data center. The DC layer includes cloud data centers and computing power belonging to various cloud providers. The DC provides long-term storage and computing power to each sector that is typically less time-sensitive. In other words, the capability of this layer would be using cloud infrastructure to run big data interactions or processes in less time-sensitive data. The main components of the DC located in the cloud network area are Data Storage, Computing power, Membership, Ledger, Smart contract, API, Miner, Consensus, Events, and Connectivity. Table 1 explains each of these key components in detail.

**Figure 2:**
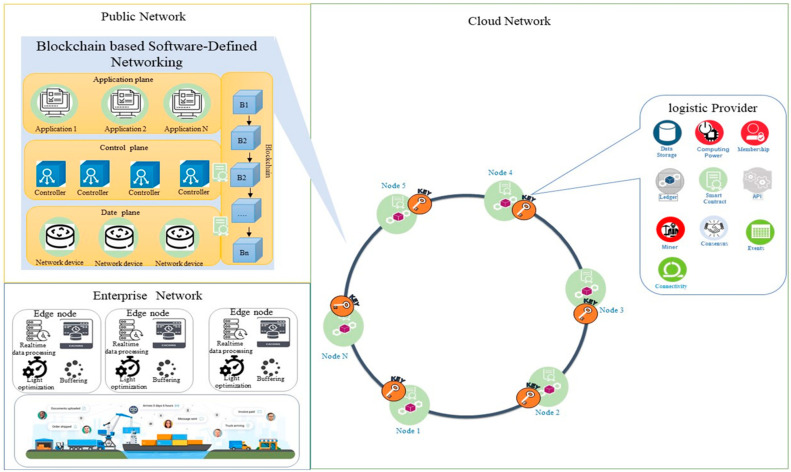
Component diagram and network specification of OL.

Figure 2 shows the key components diagram and network specification of OL. In this configuration, a logistic service provider can deliver and share assets more rapidly, reliably, and securely. The OL platform supported three types of networks. Each network is defined as follows.

1. Cloud Network: This is the high-level network that gives users access to computing resources through a provider operating inter-connected servers. This involves connecting to an enterprise blockchain network and helps distribute content quickly and securely. This network provides connectivity between miner nodes in the blockchain. Cloud service provider helps organizations deliver content more rapidly, reliably, and securely.
2. Public Network: The Public Network holds the different networks (typically the Internet), peer cloud systems, and edge services. For example, a service user uses a public network to connect to a cloud network to use services.
3. Enterprise network: The enterprise network is included in the user directory, enterprise applications, and enterprise data. For example, each distribution center has its enterprise network, which is connected to a cloud network via a blockchain-based software-defined network.

### 3.2. Blockchain-Based Software-Defined Networking Layer

Software-Defined Networking (SDN) and Network Functions Virtualization (NFV) are emerging and considered new ways of designing, building, and operating networks. These two complementary technologies can support data transition between edge nodes and cloud computing. This layer improves the dynamism of the network. For example, it is possible to change the path through which data flows between the cloud computing layer and the edge computing layer. BSDN uses a hash function instead of heavy cryptography algorithms. Therefore, BSDN can improve flexibility and performance compared to traditional SDN. This layer contains 3 planes, namely, the application plane, control plane, and data plane. It is also coupled with blockchain technology.

The application plane is an open but secure policy for various applications and devices to leverage network resources like topology and statistics. Different applications can communicate with each other and the controller via APIs and peer-to-peer communication, powered by enterprise blockchain technology.

The control plane or network brain acts as an operating system. It is a logical entity that receives instructions or requirements from the application plane and relays them to the networking components. This plane also extracts information about the network from the hardware devices and communicates back to the applications with an abstract view of the network and includes statistics and events about what is happening. The control plane system then will be programmed by the top layer using “Northbound API” or “RESTful API”.

The data plane or user plane is the part of a network that carries user traffic. It enables data transfer to and from IoT or any smart devices. Generally, it includes packet forwarding devices and communications with the network controller.

The blockchain is used as a service that provides functionalities of resource tracking and asset sharing among multiple controllers on the control plane. Therefore, resource tracking is used to track and trace the network resources of each controller and asset.

### 3.3. Edge Layer

The edge layer provides real-time connectivity and limited computing power, representing the perspective of industrial devices. It brings memory and computing power close to the needed industrial device. Edge nodes represent gateways and data capturing services. This layer is where the cloud resources are being distributed and moved near to the end-users and end devices. The reasoning for using edge nodes is to evaluate time-sensitive data closer to the location where data flows are collected, hence taking some of the computational load off the resources at the cloud computing layer and decreasing network load. In an early warning system, these nodes consist of services to receive data over an explicit link from devices, filter the input data stream, aggregate the measured values and send the data to the cloud computing layer. Hence, an edge computing layer can reduce significant traffic from the core network. That is why OL provides low latency and location awareness and optimizes users’ experience under quality-of-service requirements for time-critical and even real-time applications. So, the OL has the characteristics of decentralization and real-time change and involves networking and the mechanism of communication between nodes.

### 3.4. Device Layer

The device layer or IIoT layer provides ubiquity connections with industrial devices or service users. This layer is responsible for data transferring between the drives (machines and robots) and the service providers in a real-time manner. Physical components such as a robot, truck, or trailer can remotely control or send and receive data. IoT devices are connected with an autonomous agent. This node connects to OL via membership verification. This connection is long-lasting, and each node needs membership verification; this connection is explained in reference [28].

## 4. The Key Characteristics of OL

In this section, the key components of the OL are presented and discussed in detail. Firstly, we explain the blockchain asset sharing service and propose a proof of delivery concept based on a smart contract.

### 4.1. Blockchain Asset Sharing Service

**Figure 3:**
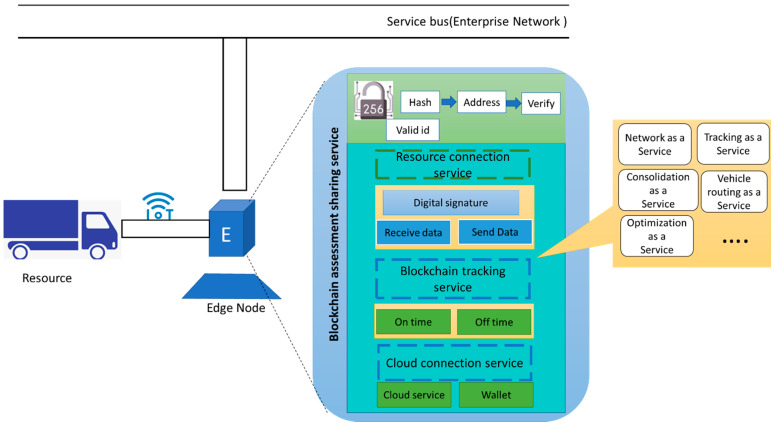
Blockchain asset sharing service: The driver can use a mobile application to connect OL.

As shown in Figure 3, the structure of the blockchain asset sharing service (BASS) is presented. BASS has three main responsibilities. First, it provides connectivity and data integration between the cyber and physical environment. Secondly, it is responsible for tracking and tracing the resource. Therefore, the resource owner and services requester can have tracking information. The resource tracking facilitates the pay-as-you-go method for each service and also improves the trust between parties. Thirdly, BASS provides a real-time connection with the distributed cloud layer via BSDN; it has two modules, including cloud service and wallet. Cloud service communicates with the cloud network and sends/receives transactions to/from the network. Connecting with the cloud environment enables cloud services, for example, the vehicle of carriers that can use vehicle routing as a service. Also, each node has a wallet on the blockchain network. The potential transaction/messaging services need to use this wallet account.

### 4.2. Smart Contract and Proof of Delivery

**Figure 4:**
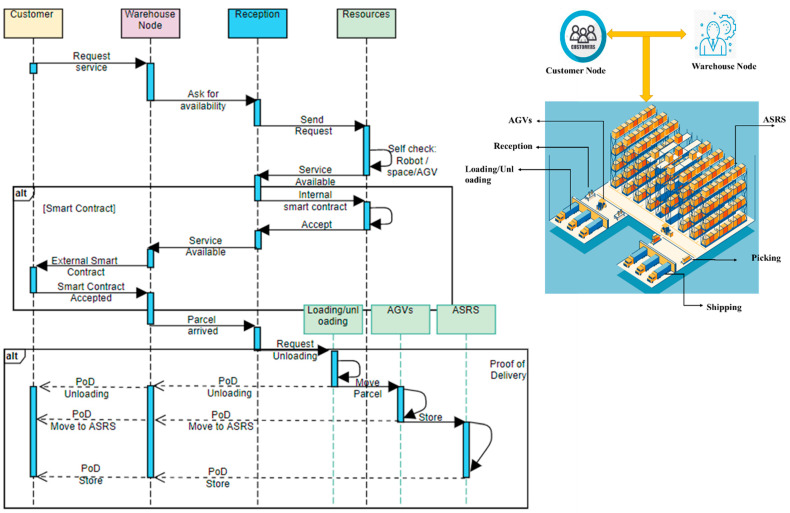
Process of requesting service from the distribution center.

The proof of delivery (PoD) is the foremost final step that should perform by the service provider. PoD is still one of the most complicated problems even today. The impact of PoD delays and errors is even doubled due to COVID-19. This error has two significant consequences on the sharing platform. First, having dissatisfied customers or losing their trust, and second, it causes a substantial delay in financial terminations. However, smart contract functions on the OL improve the trust and provide PoD between all steps of resource sharing. Those nodes that wish to obtain any logistic services from the platform use a smart contract to send a request, then the service provider accepts this service via smart contract; then, after performing each service, the PoD of service sends to the customer. This process is explained via a sequence diagram for renting inventory space in Figure 4. Customers first request a service (store parcels in the warehouse). The distribution center node receives this request and asks if it is possible to perform this service using sub-contractors (assets and resources), if subcontractors can perform this service, if they can send service availability, and establish an internal smart contract between them. Then the distribution center initiates an external smart contract with the customer after the customer accepts the smart contract. The service is available to the customer; in this example PoD of each service send to the distribution center node and customer node. This process is done by the blockchain track service module in the BASS. Therefore, the distribution center (service provider) and customer (service user) can track each process and resource.

In this method, we have two levels of a smart contract. An internal smart contract providing a contract between internal subcontractors (assets and resources); for example, in the distribution center; this contract applies between reception, loading/unloading, and AGV, or AGV, and ASRS. Second, a smart contract which is an external contract to be applied between the customer node and distribution center node. The PoD process is illustrated in the second section of Figure 4 for loading/unloading resource sharing.

### 4.3. Mechanism of Open Logistics Platform

**Figure 5:**
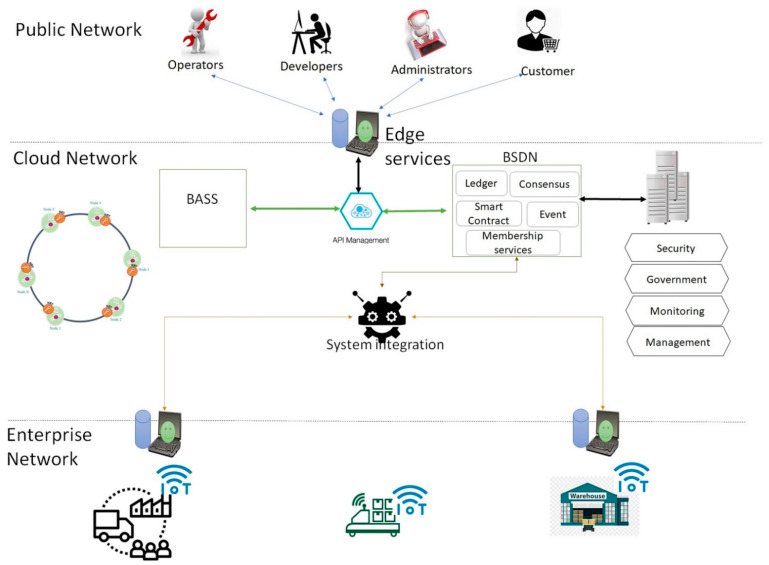
Typical capabilities needed for customers or service providers.

As a main mechanism of the OL, customers use the public network and public keys to sign and send a request. Therefore, the transaction tracks via the user’s public key and the digital signature also improve security and data integrity in the system. Then, the transaction broadcasts to a one-hub neighbor of the node. After that next node which is the neighbor node verifies the broadcast transaction obeying the transaction protocol and broadcasts it to the next node, or the transaction will be dropped. Afterward, all transactions created by the network through an agreed period are bundled into one block by a mining node. The mining node broadcasts the block to OL. Then, the receiver miner node will confirm all the transactions in blocks, and they need to obey the transaction protocol. The current block has a hash of the previous block. Therefore, if the block passes the verification, the receiver adds it to the blockchain and extracts the transactions it contains to be updated. Otherwise, the block is discarded. To achieve this mechanism, Figure 5 depicts the typical capabilities of the OL. It is articulated based on three networks, including public, cloud, and enterprise.

A cloud network is responsible for supplying unlimited blockchain-based services to users and service providers. API management publishes lists and updates APIs in a wide variety of deployment environments. This helps contractors and customers rapidly gather results through the discovery and reuse of the existing assets and resources. BSDN should interface with the blockchain network as part of its operation. It achieves by using the interfaces provided by the miner node. BSDN supports essential capabilities for blockchain solutions in a node. While each miner node is set up and implemented differently, these core capabilities should be considered in BSDN and solutions. It consists of a ledger, consensus algorithm, smart contract membership services, and event management.

Systems integration is a feature of integrating platforms, including API adapters and enterprise service bus (ESB) connections between the blockchain platform and the enterprise systems. The enterprise network is comprised of the enterprise user directory, enterprise applications, and machine data. Machines or devices data includes metadata and systems of record for enterprise applications. Machine data can stream immediately to data integration or the data repositories, which provides a feedback loop in the analytic system.

## 5. Simulation Platform

**Figure 6:**
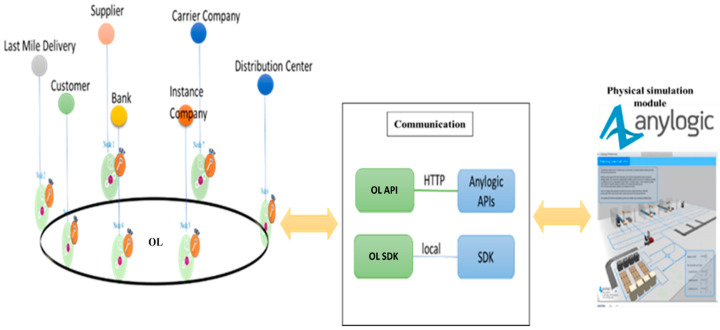
Develop a simulation platform.

The resource-sharing platform involves a significant number of different resources and assets which are associated with various third-party service providers (3PL), such as trucks, trailers, material-handling systems, and storage systems. Analyzing this type of system in a real environment is infeasible because of the long period that would be required for the development of resources for each service. It is also very difficult to test the multiple scenarios when attempting to compare alternative solutions. One way of overcoming this problem is to use a simulation platform that behaves like a real system. We developed a simulation platform to evaluate the potential benefits of employing OL. Figure 6 illustrates the developed simulation platform with its three main components. The main advantage of this simulation platform is that it can distinguish the software level from the hardware level. Therefore, as with the real implementation, each service has three different entities in this simulation platform: (1) the agent inside the physical simulation module, which simulates the capability of the hardware and local decisions such as a vehicle, ASRS, robot, etc. (2) the communication channel for each specific service which is responsible for transaction between OL module and physical simulation module. (3) the OL module is an implementation of the proposed platform.

### 5.1. Physical Simulation Module

In this section, we consider a process of sharing truck and swap drivers in the transportation hub as a case study. Logistics resource (e.g., truck and drivers) sharing between service providers has been common in recent years. Still, different companies make different decisions about providing logistics services or sharing. We consider three suppliers with multiple 3PL service providers. After the performance of each service, the PoD will be sent to the service requester and service provider. The most important information between supplier (service user) and 3PL service provider is defined in Table 2. This physical simulation module simulates product delivery in the USA. The supply chain includes four manufacturing facilities and ten distribution centers that order a random amount of the product every 1–3 days. Three 3PL carrier companies provide delivery services between the manufacturers and distribution centers. When a manufacturing facility receives an order from a distributor, it checks the number of products in storage; then, it signs the smart contract with the distributor if the required amount is available. Then it sends a delivery request to carrier companies. The first company to complete the mining task will be awarded a delivery contract. The reward carrier company signs the smart contract with the manufacturer to perform the service. We consider four 3PL service providers for the last mile delivery task. So, when the customer purchases the product, the nearest distribution center receives the request, then checks the product availability. If the requested product is available, then the distribution center sends a last-mile delivery request to service providers. The first last-mile delivery company which completes the mining task will be awarded a last-mile delivery contract. The rewarded last-mile company signs the smart contract with the distribution center to perform the service. All PoDs of services are then shared with service users and service providers.

This model is essentially a multi-model. Distributors, 3PL, trucks, resources, and manufacturing facilities are agents with customer behavior. Agents live in a GIS (Geographic information system) space. GIS search engine is used to find locations on the map and place agents there. Trucks move on real roads, and routes are created when vehicles start moving to destinations. Customers located near each distribution center, considering a maximum five-hour distance.

The carrier service is performed by truck with 1000 product capacity from supplier to distributor, which takes 6 h (the average time of all drives). Each loading and unloading process action needs 20 min (the average time of all processes). For each product, the processing time for packaging and shipping was 10 min, and the queue time for the AVG in the line was three minutes. Each product was assumed to be sent to a different destination and required a separate packaging processing time. The last-mile delivery time for each package was normal distribution with a mean of 4 h and a standard deviation of 5. And we consider 5000 parcels for each day needed to deliver to the customer. The capacity of a truck for last-mile delivery is 500 products, and we assume this number is fixed for all last-mile delivery services. The tests considered five days of working hours. The adapted time unit was 1/150th of a minute, the standard time data provided.

### 5.2. Implementation of OL

**Figure 7:**
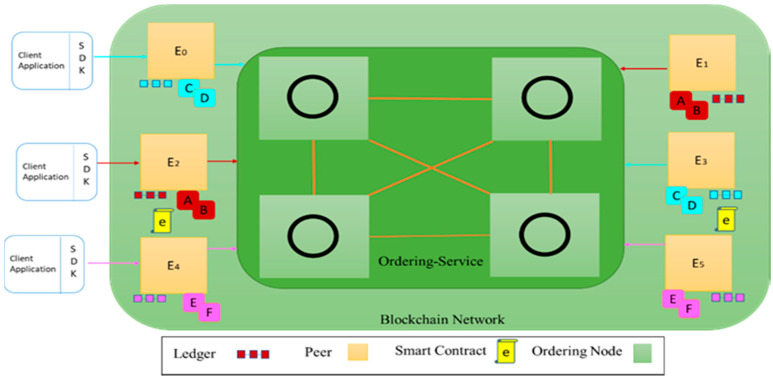
Developed blockchain network: three clients and 6 edge nodes.

To develop OL by Hyperledger Fabric, we set up a multi-host blockchain network with three physical machines. We created OL with one Orderer organization and six peers’ organization. Figure 7 shows the developed blockchain network. Peers E0 and E3 connect to the Cyan channel for chain codes C and D, and peers E2 and E1 connect to the Red channel for chain codes; A and B, and peers E4 and E5, connect to the Pink channel for chain codes E and F. We implemented a smart contract with a following endorsement policy appropriate to peers. We also implemented practical Byzantine fault tolerance (pBFT) as a consensus algorithm.

#### Implementation of Proof of the Delivery and Smart Contract

The proposed PoD system contains two main elements Nodes and Smart contracts. Each element is explained as follows:

Node: A user who uses a mobile, web application, or IoT to connect OL via a public network or enterprise network. We clustered this user into two main types, namely user node (UN) and provider node (PN). The node that requests a service is called a UN. Any participant who will accomplish the service by fulfilling all the requirements is called a PN. All the nodes will have to take the authorization process to utilize the system and have a user interface to update their capabilities. This process is accomplished by registering themselves in the system. each node has the following attributions:

- N is the unique node ID
- Loc_N_ is the location of the node on the map (attitude and longitude).
- T_N_ is the latest time at which the node participated in any services.
- S_N_ is the status of the node (e.g., busy, or available).
- Cs_n_ is a class of service provided to OL by node NP.
- Cr_n_ is a class of service request by Node NU.
- Ss_id_ is a unique id for each service in the system.
- Cp_id_ is the current satisfaction point of the service.
- Loc_id_ is the location where the service needs to be performed.
- R_id_ is a unique resource or assets id (truck ID or drivers ID) which performs the specific service and corresponds to the BASS.
- Ra_id_ is the status of a resource that performs a specific service.
- Loc_R_ is the location of the resource that performs the service.

The UN can request service at the OL respective to time, location, and cost. The UN must provide the service location, the due date of service, number of needed services and budget for service, service type (e.g., last-mile delivery, distribution center), service description, and minimum requirements of the NP to perform this service. A smart contract is an agreement between the UN and NP. The contract runs automatically according to predefined rules and protocols. Algorithm 1 shows the specific action of the smart contract between UN and UP, which is named an internal contract. The output of this algorithm is a contract between NUs.

**Algorithm 1.** Smart contract between UN and carrier NP**Input:** UN, UP, S_n_, Loc_N_**Output:** The updated contract state between NU and NP1: **if** (Cr_n_ == Cs_n_)2:  if (S_n_ == available && Loc_id_ == Loc_R_) **then**3:   **if** (Ss_id_ != create) **then**4:    Let Ss_id_ = ServiceToBeCreated.NP;5:    NP initiates to create 6:    Request sends to NP;7:   **else if** (Ss_id_ ==create && accepted by NP) **then**8:     Ss_id_ update & service sent to UN by NP;9:   **else if**10:    Show an error and update contract state11:   **else**12:    Show an error and update contract state13:   **end**

Algorithm 2 shows the internal smart contract inside NP between manager and resources.

**Algorithm 2.** Smart contract for internal NP and update ststus to NU**Smart contract 2:** Resource state for internal- NP**Input:** Ra_id_, NP, S_n_, Loc_R_, Ss_id_**Output:** The updated contract state of newest resource state1: **if** (NP == NP1) **then**2:  **if** (Ra_id_ != Updateby Ss_id_) **then**3:   **if** (outcome== True) **then**4:    Let Ra_id_ = Intra- Ss_id_5:    Create and send a notification about the start of the service.6:   **else if**7:    Next step cannot start, update status to the system8:     **else if**9:    Show an error and update contract state10:  **else**11:   Show an error and update contract state12: **end**

In this study, all chain code (smart contract) is written in the GO language, and the OL mainly permits the authorized node to operate the system. To request a service or to receive a service. After completion of the registration process, the OL will provide a unique user identity (User ID) to the user. However, there might be several malicious users who can exploit the system by creating fake user identities. This kind of intrusion is called the Sybil attack. To prevent a Sybil attack, the participant should submit an identification card and must be approved by the other members of the OL.

### 5.3. Communication Module

**Figure 8:**
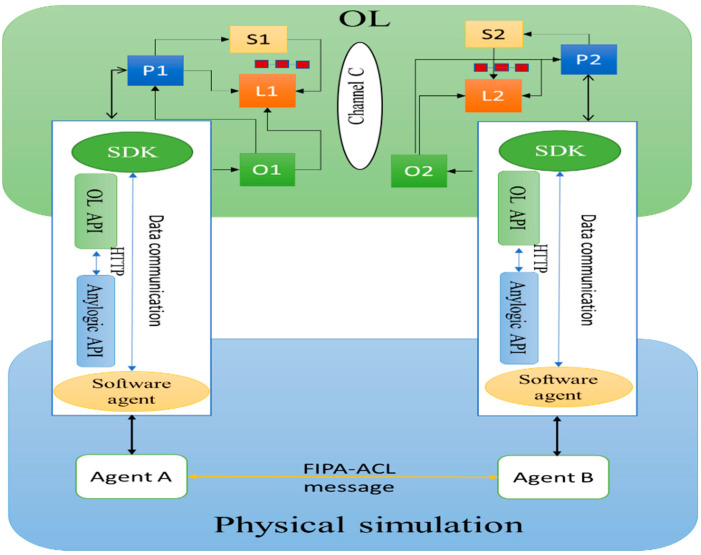
Communication between physical simulation module and OL.

This module is responsible for providing the interaction between the agent in the physical simulation module and OL. Figure 8 shows how agents in the physical module interact with peers to retrieve the ledger in the OL module. Ledger request connections involve a simple three-step dialogue between an agent and a peer. Ledger update interactions are a little more involved and require two extra steps. Agents connect to peers when they need to access ledgers and smart contracts. Via a peer connection, agents can execute smart contracts to query or update a ledger. The result of a ledger query transaction is returned immediately, whereas ledger updates involve a more complex interaction between agents, peers, and orderers. In Figure 8, agent A links to P1 and evokes smart contract S1 to inquire or update the ledger L1. P1 evokes S1 to create a response proposal that contains a query result or a ledger update. When agent A gets the response, then the process is completed. After that, for updates, agent A creates a transaction that contains all responses, then transmits it to O1. O1 accumulates transactions through the network into blocks and distributes these to all peers. When transactions are sent to P1 via O1, P1 validates the transaction, then applies to ledger L1. Once L1 is updated, P1 generates an event received by A to signify completion.

### 5.4. Results and Discussion

For evaluation and validation of OL based on the case study, we first investigate the result of the case study, then test the performance of the core OL network.

#### 5.4.1. Result of Case Study

We evaluated the case study based on the two main KPIs: response time (RT) and service usage rate (SUR). RT is the total amount of time it takes to respond to a request for service. RT represents the response time between the service requester and 3PL. We compute the RT from the request initialization until 3PL accepts and signs the smart contract. In this step, we simulated for five days and collected data for 5000 daily orders. The transaction size, block size, and response time are shown in Table 3. We can highlight two key results from this table. First, the transaction size and block size directly affect the RT. Second, the block size of service for the internal distribution center is much larger than other services because we considered all services in one block, and multiple smart contracts need to sign simultaneously.

**Figure 9:**
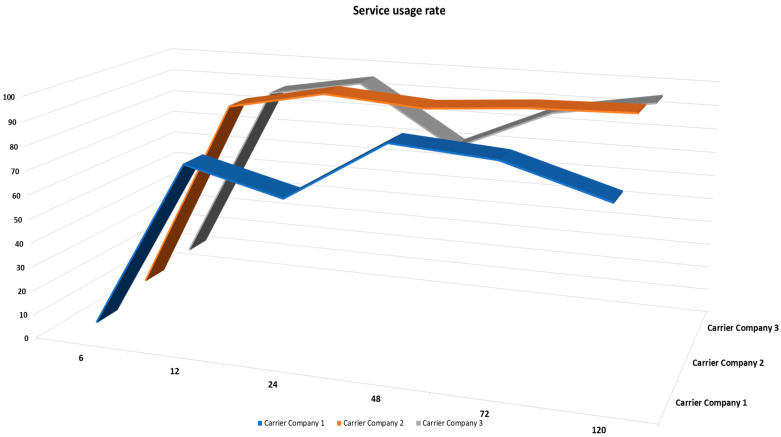
Service usage rate between the distribution center and manufacturers: three carriers’ companies.

SUR represents whether the manufacturer or distribution center can control the requested service (resource sharing). If the utilization rate is high, the service requester can communicate with the 3PL in time and obtain updated information. If the information-sharing efficiency is low, the service requester cannot know in time which 3PL has the idle resources. As a result, manufacturers or distribution centers cannot make immediate arrangements, which may hold products in manufacturers or distribution centers for a long time. For the 3PL, if the manufacturer or distribution center fails to request service in time, resulting in a low SUR, it will reduce the economic efficiency of the 3PL. For the 3PL, when the manufacture or distribution center still needs service, but the SUR is still low, the manufacture or distribution center and the 3PL have low information sharing capacity and low communication efficiency, which will also reduce the cost economic efficiency of the 3PL. The statistical results are shown in Figure 9. The result shows that the SUR is very low at the first 6 h, but after 12 h, the SUR normally is more than 80 percent. This happens because we didn’t consider the warming time in the system.

#### 5.4.2. The Core OL Network Evaluation

**Figure 10:**
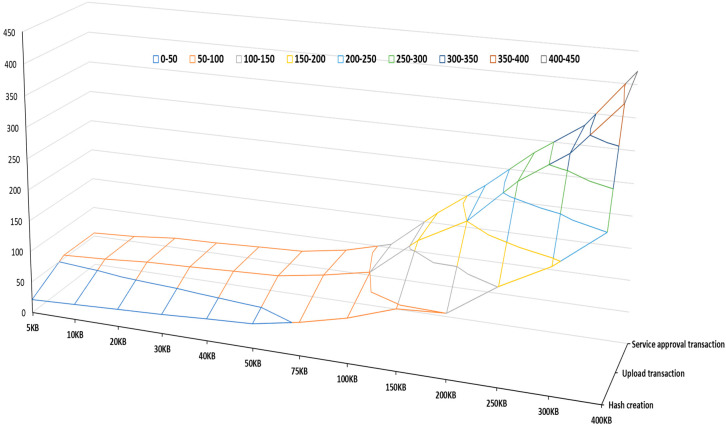
Surface chart of the latency of various transaction sizes: 9 different ranges.

The performance analysis of the core OL network was conducted to evaluate the impact of transaction size on transaction response times, known as latency. Latency is one of the main key KPIs to evaluate the performance of the blockchain network in the platform. We consider three types of transaction time to deliver a wide-ranging assessment of latency, hash creation time, transaction to block via agents, and service endorsement time. Figure 10 presents a surface chart of the latency of various transaction sizes, ranging from 10 kB to 400 kB. We consider this range because it can support all transactions in the OL. It proves the trend that the total time increases with the transaction sizes. Moreover, the increasing rate enlarges when the total transaction size is over 150 KB, which provides insights for block size configuration to enable an OL with high performance.

There are three key points in this chart.

I. It clears that the size of the transaction is related to the time of the transaction. A larger transaction indicates a longer hash creation time, upload transaction to block via agent time, and service approval transaction time. The results show that transaction size influences the standard deviations of times, which generates a greater impact on upload transactions to block time.
II. A huge difference exists between 100 KB and 150 KB transaction sizes, showing that the network will face a latency problem if the transaction size is more than 150 KB. So, the best transaction for the OL is between 5 KB to 100 KB.
III. The total time of the transaction for block sizes 300 and 400 is quite high. This rise in transaction time creates instability in the system. Therefore, each operation concerning a transaction size greater than 250 KB brings unpredictability to completing the requested operation. Therefore, we suggested setting the block size as 150 kB to guarantee a stable and high performance in the OL.

**Figure 11:**
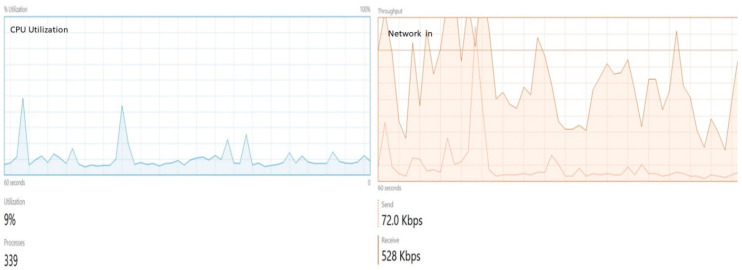
Monitoring network: left side figure shows CPU utilization, and the right side shows network in results.

Figure 11 denotes the monitoring data. In this figure, we show the results of two main KPIs of the OL environment. These indicators include CPU utilization and network in. These results show that the OL can support real applications without status failed issues and require less CPU utilization. Therefore, the developed network has an acceptable level of scalability for big data support.

**Figure 12:**
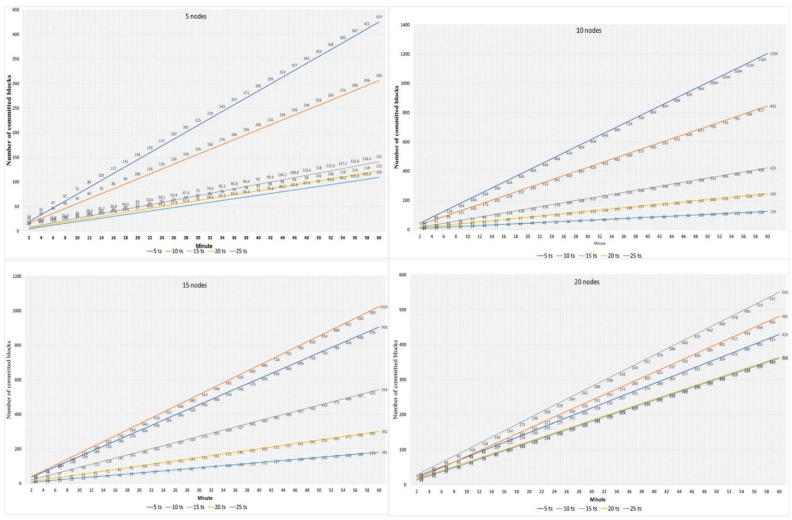
Block committed per minute: with various sized blocks (5 nodes, 10 nodes, 15 nodes, and 20 nodes).

We recorded the network operation of the number of committed blocks every minute for all experiments. Figure 12 shows the number of committed blocks for different block sizes. We categized these results by considering the different numbers of nodes in the network 5, 10, 15, and 20, respectively. The number of transactions is 5, 10, 15, 20, and 25 per second. The result shows that the network’s performance is reduced by increasing the number of transactions. Nevertheless, when increasing the number of nodes to 15 and more, the network performs differently. With 15 nodes block committed to 10 ts is more than 5 ts. For example, in the first 10 min of simulation, 175 blocks are committed to 10 ts transactions, a number 20 more than 5 ts. Better performance is observed when committing 20–25 transactions to a block than when committing 5–10 transactions to a block for the network of 20 nodes. The frequency of generating new blocks is inversely related to the block size if a small block size and a constant transaction generation period are used. Increasing the frequency of block generation leads to an increase in the number of transactions within the protocol, most of which are sent from each node to all others. As a result, the network becomes overloaded, reducing performance and leading to a decrease in the number of committed blocks.

#### 5.4.3. Qualitatively Analysis

The proposed platform is compared qualitatively with two main existing platforms, including traditional sharing and web-based sharing platforms. According to the existing definitions for sharing platform characteristics presented in the literature, some common key characteristics, such as scalability, that is, the ease to use or customize and learnability, privacy, tractability, and security, are selected and compare the proposed OL with the other existing platform. Besides, some criteria reflecting the cost are also illustrated, such as transaction speed. The result of this comparison is shown in Table 4. It is clear that the OL, due to the advantages of blockchain-enabled technology and a smart contract-based service sharing model, could provide better security, liability, and transparency to all stockholders, which improves the trustability of the whole system.

## 6. Conclusions and Discussion

Asset and resource sharing among partners in a supply chain is commonly considered a key factor in enhancing supply chain performance. Sharing logistics improves efficiency at delivery points and cuts transport costs and environmental impact. However, due to the lack of trust and arising security issues of shared resources, the companies didn’t rely on the third-party services provider.

The open logistic network platform can speed up the delivery cycle and reduce the associated cost of delivery and product storage. On the one hand, trustworthy and transparent service sharing can effectively use the slack supply chain resources to increase the utilization of the facilities and manpower of the supply chain and logistic systems. On the other hand, secure data sharing among companies can help them focus on their core competencies. In addition, blockchain and smart contract asset sharing mechanisms enable a secure and standardized approach to achieve a higher level of sharing among stockholders. Furthermore, the edge computing approach improves flexibility and brings extra distributed networks to the supply chain environment, which is highly aligned with SC4.0.

In this paper, we attempt to solve the main problems of sharing resources and assets in logistics. We propose a novel blockchain-enabled resource and asset sharing platform for realizing data exchange and service sharing in logistics based on blockchain and edge computing technology and named it OL. Firstly, we provided the OL computer and network architecture, then the key components and mechanism of OL were explained in detail. The proposed platform supports an open, decentralized, yet secured network and a new type of proof-of-delivery for transparency of resource sharing. An experimental simulation was conducted in the context of B2C logistics, and the feasibility of the proposed platform was evaluated via different and suitable KPIs.

The expected benefits from the OL platform include: (1) The new PoD based on the smart contract helps the 3PLs effectively track and trace their resources and assets, easily improving trust, payment liability, and insurance problems. (2) The OL platform, with the help of AI, would improve information security and a trustworthy connection to IoT devices. (3) The OL platform would provide the distributed computing power, which is interoperable with the enterprise and public network.

## Figures and Tables

**Table 1:** Explanation of each DC components.

Name	Definition
Event	Events are notifications of considerable modifications or actions that arise in the network, like the performance of a smart contract or the formation of a block.
Membership	It manages uniqueness, privacy, confidentiality, and auditability on the network.
API	It is a typical integration method that defines interactions of adapters, enterprise service buses, and multiple software between the blockchain network and the SMEs.
Smart contract	The smart contract is a computer program executed in a secure environment within the blockchain network. Smart contracts encapsulate business logic involving contract terms and conditions between agreeing participants. In the proposed platform, three different types of smart contracts exist, which will be explained in the next section.
Ledger	It is a sequence of cryptographically linked blocks that contain transactions.
Consensus	A process used by the nodes in the network to agree on the legality and validity of transactions appended to the ledger. This process maintains a consistently replicated ledger within the network.
Connectivity	This segment allows reliable and secure connections to systems and the ability to filter, aggregate, or change data among cloud and blockchain components and enterprise systems.
Miner	Miner is responsible for adding transaction data to the ledger of past transactions. In the ledgers, blocks are secured by miners and are connected to form a chain.
Computing power	Computing power will play the role of the engine and provide all related computing power to the system, such as mining power, data power, etc.
Data Storage	Data storage is responsible for collecting and storing digital information—the bits and bytes behind applications, network protocols, documents, etc.

**Table 2:** Information between supplier (service user) and 3PL service.

	Information Sends to OL	Information Retrieves from OL
Manufacture or supplier	Number of products Status Destination Address of Supplier ETA/ETC Id of product Weight of product Size of product Type of services	Available services Schedule ETA Resource ID Company ID PoD Smart Contract for each service Track Service Track Product Emergency Number External smart contract Status
3PL	Available resources ID of resources Company location Available services Statue Type of services	Available service request Id of service Location of requested service Internal smart contract PoD Track service Track parcel Track resources Status Schedule ETA Routing service

**Table 3:** Response time of the system.

Service Requester	Service Provider	Type of Service	Size of Transaction	Block Size	Response Time (Mean)
Distributor	Manufacture	External: Request product	16 bit	40 tps	210.9
Manufacture	3PL/carrier companies	External: Request delivery service to the distribution center	32 bit	60 tps	345.4
Distribution center	Distribution center	Internal: request internal service	128 bit	120 tps	648.3
Distribution center	3PL/last-mile delivery	External: Request delivery service to end customers	64 bit	80 tps	415.9

**Table 4:** The typical comparisons with the existing and currently used platform in qualitative method.

Sharing Platform	Traditional Sharing Method (Long Term Contract)	Collaborative Platform (3PL Web Based Platform) [29]	Cloud Based 3pl Platform [30]	Public Blockchain [31]	Open Logistic
Scalability	✓	✓	✓	✓	✓
Privacy	✓	✓	✓		✓
Security	✓				✓
Transparency			✓	✓	✓
Trust-ability					✓
Liability				✓	✓
Decentralization				✓	✓
Interoperability			✓	✓	✓
Pay-per-use			✓	✓	✓

## Data Availability

Not applicable.
